# A new genomic tool for walnut (*Juglans regia* L.): development and validation of the high‐density Axiom™ *J. regia* 700K SNP genotyping array

**DOI:** 10.1111/pbi.13034

**Published:** 2018-12-04

**Authors:** Annarita Marrano, Pedro J. Martínez‐García, Luca Bianco, Gina M. Sideli, Erica A. Di Pierro, Charles A. Leslie, Kristian A. Stevens, Marc W. Crepeau, Michela Troggio, Charles H. Langley, David B. Neale

**Affiliations:** ^1^ Department of Plant Sciences University of California Davis CA USA; ^2^ Research and Innovation Centre Fondazione Edmund Mach San Michele all'Adige TN Italy; ^3^ Department of Evolution and Ecology University of California Davis CA USA

**Keywords:** Persian walnut, genomic variants, SNP array, molecular breeding, pedigree, genetic diversity

## Abstract

Over the last 20 years, global production of Persian walnut (*Juglans regia* L.) has grown enormously, likely reflecting increased consumption due to its numerous benefits to human health. However, advances in genome‐wide association (GWA) studies and genomic selection (GS) for agronomically important traits in walnut remain limited due to the lack of powerful genomic tools. Here, we present the development and validation of a high‐density 700K single nucleotide polymorphism (SNP) array in Persian walnut. Over 609K high‐quality SNPs have been thoroughly selected from a set of 9.6 m genome‐wide variants, previously identified from the high‐depth re‐sequencing of 27 founders of the Walnut Improvement Program (WIP) of University of California, Davis. To validate the effectiveness of the array, we genotyped a collection of 1284 walnut trees, including 1167 progeny of 48 WIP families and 26 walnut cultivars. More than half of the SNPs (55.7%) fell in the highest quality class of ‘*Poly High Resolution*’ (PHR) polymorphisms, which were used to assess the WIP pedigree integrity. We identified 151 new parent‐offspring relationships, all confirmed with the Mendelian inheritance test. In addition, we explored the genetic variability among cultivars of different origin, revealing how the varieties from Europe and California were differentiated from Asian accessions. Both the reconstruction of the WIP pedigree and population structure analysis confirmed the effectiveness of the Applied Biosystems™ Axiom™ *J. regia* 700K SNP array, which initiates a novel genomic and advanced phase in walnut genetics and breeding.

## Introduction

*Juglans regia* L., also known as Persian or common walnut, is the only species of the genus *Juglans* widely cultivated for nut production (Jain and Priyadarshan, 2009). Currently, Persian walnuts are grown in widely distributed temperate regions extending from North, Central and South America to Europe and North Africa, through Central Asia from the Caucasus to China and in Oceania (FAOSTAT data, 2016). China is currently the world's leading producer, while the United States dominates the global export market (USDA‐Foreign Agricultural Service). Overall, worldwide walnut production has grown by almost 250% over the last 20 years (FAOSTAT statistics, 2016), most likely a result of increased regular consumption of walnut due to its numerous benefits to human health (Costa *et al*., 2014).

Genetic improvement of walnut only began in the 20^th^ century (McGranahan and Leslie, 2012), and today the most important walnut breeding programs worldwide are located in California, France, China and the Middle East (Bernard *et al*., 2018). In California, which accounts for 99% of the US walnut production, the Walnut Improvement Program of University of California, Davis (UC Davis WIP) was established in 1948 under Eugene F. Serr and Harold I. Forde to develop improved cultivars for California growers (Leslie and McGranahan, 2014). The UC Davis walnut germplasm collection, which contains wild accessions, established cultivars and advanced selections, as well as representative genotypes from other countries, (e.g., France, China, Afghanistan and Japan), has served as a *reservoir* of materials for the breeders of the WIP (Tulecke and McGranahan, 1994). Enhanced yield, extra‐light kernel colour, late leafing and early harvesting dates and increased disease resistance are the main goals of the UC Davis WIP (McGranahan and Leslie, 2012). In this regard, French cultivars, donors of excellent kernel qualities and late leafing and Californian varieties with precocious and lateral fruit‐bearing, have been used as parents of controlled crosses in the WIP, especially during its first phase from 1948 to 1979. Over the years, the UC Davis WIP has released more than 20 new cultivars for the walnut industry, including the most popular ′Chandler’, which now comprises over 53% of California walnut production (California Department of Food and Agriculture, 2017) and 75% of nursery sales (California Agriculture Statistics Service, 2016).

To date, the WIP has been based on controlled outcross pollination and phenotypic selection over several generations. However, phenotypic selection can be inefficient, notably for complex, polygenic traits. Moreover, the long juvenile phase for walnut, which lasts from 3 to 9 years, extends the release of new cultivars able to respond to uncertain climate change and the evolving preferences of the walnut industry (Nocker and Gardiner, 2014). In the current genomic era, transition from conventional to genome‐assisted breeding is fundamental to increasing genetic gain through more accurate selection of superior genotypes and accelerating the development and release of new cultivars. The discovery and large‐scale genotyping of thousands of genome‐wide molecular markers are the first steps in this transition to molecular breeding (Sehgal *et al*., 2016). In walnut, most genetic studies have been based on a handful of molecular markers, notably Random Amplified Polymorphic DNA (RAPD–Nicese *et al*., 1998; Woeste *et al*., 1996) and Single Sequence Repeats (SSRs–Pollegioni *et al*., 2017; Woeste *et al*., 2002). However, these types of molecular markers are laborious, time‐consuming, not evenly distributed across the genome and, therefore, of limited use in implementing new genome‐based approaches such as genome selection (GS) and association mapping (AM; Rasheed *et al*., 2017). Single nucleotide polymorphisms (SNPs) are better candidates for crop molecular breeding due to their abundance in plant genomes and the development of cost‐effective and high‐throughput SNP genotyping methods such as array‐based technologies (Ganal *et al*., 2012). You *et al*. (2012) identified 22 799 SNPs in bacterial artificial chromosome end sequences (BESs) of the walnut cultivar ‘Chandler,’ of which 6000 were selected to generate a 6K Infinium SNP array. However, this SNP genotyping platform is based on only the genetic variability of ‘Chandler,’ which can be quite limiting for broader genetic studies.

The release of the first reference genome sequence in walnut offers the opportunity to align sequences from different walnut genotypes and identify variation in their genome sequences (Martínez‐García *et al*., 2016). Recently, Stevens *et al*. (2018) reported the whole‐genome resequencing of samples from four *Juglans* species to capture and compare nucleotide diversity at the inter‐species level. In Persian walnut, they re‐sequenced 27 accessions and discovered over 9.6 m SNPs. We utilized this set of filtered genome‐wide SNPs to study the intra‐species variation of Persian walnut and build the new Applied Biosystems™ Axiom™ *J. regia* 700K SNP array, currently the largest SNP chip for a tree crop. In this paper, we provide more details on the re‐sequencing panel and describe the procedure of SNP filtering and selection for the development of the Axiom™ *J. regia* 700K SNP array. Moreover, we discuss its application in reconstructing the WIP pedigree as a preliminary step towards full application of molecular breeding of walnut in California, and its use in examining the genetic diversity of germplasm collections. The Axiom™ *J. regia* 700K SNP array represents a landmark for walnut genetics and breeding, which now enters a new phase where advanced genomic approaches, such as AM and GS, can be implemented in support of conventional breeding methods.

## Results and discussion

### SNP selection for the Axiom™ *Juglans regia* 700K array

The initial step in the transition from conventional breeding to genomics‐assisted breeding is the discovery of DNA sequence variation. Stevens *et al*. (2018) re‐sequenced 27 walnut accessions selected as either representing the UC Davis WIP founders or showing interesting phenotypes for traits of interest in walnut (e.g., kernel colour; Martínez‐García *et al*., 2017). The discovery panel is mostly comprised of US accessions, but also includes wild germplasm and cultivars introduced to the UC Davis WIP from other leading countries in global walnut production, namely China, France, Afghanistan, Japan and Bulgaria (Table S1). The depth of coverage expressed as the number of equivalent genomes (X) was high (70X to 90.5X, average 73.2X), and the percentage of unmapped reads was in all cases lower than 1.7%. In addition, the mean depth of genome sequence coverage was higher than 68 for all the re‐sequenced individuals, assuring high reads counts of actual variants in all genotypes (Table S1).

**Figure 1:**
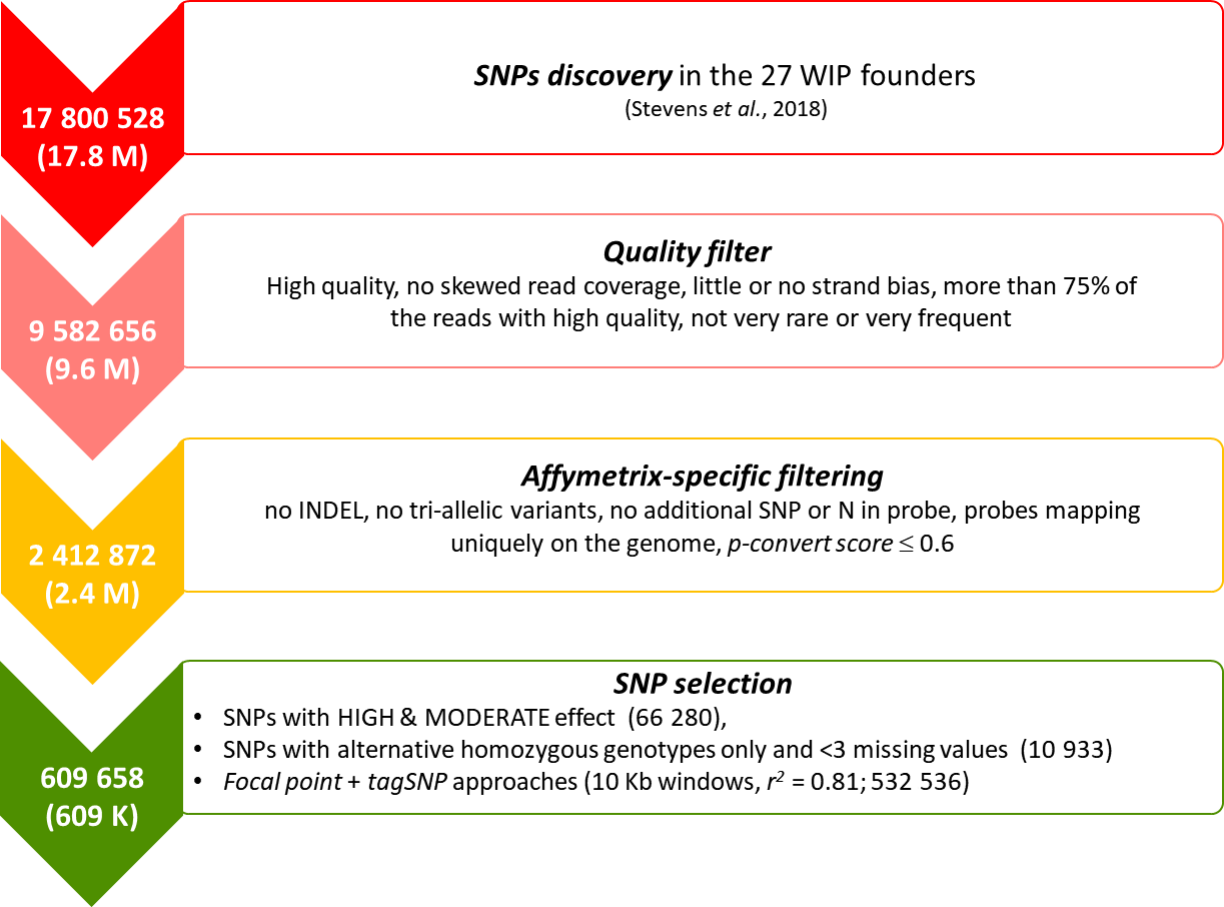
Pipeline of SNP detection and selection for the Axiom™ 700K *J. regia* array.

Based on the mapped reads, 17 800 528 SNPs distributed over 9042 scaffolds were identified (available at https://www.hardwoodgenomics.org/Genome-assembly/2209433). The number of SNPs was then reduced by almost 50% (9 582 656 variants) after applying quality *filters* (Figure 1; Stevens *et al*., 2018). Following Affymetrix (now part of Thermo Fisher Scientific, Waltham, MA) recommendations (see Figure 1), we selected 2 412 872 SNPs potentially suitable for incorporation on the SNP array, by discarding polymorphisms with complex genotypic patterns (i.e. indels and tri‐allelic SNPs) or with a high likelihood of producing incorrect or unspecific genotypes (i.e. entries adjacent to highly repetitive regions and variants with a poor Affymetrix conversion score).

The subsequent selection of SNPs for inclusion on the array focused on two main aspects: (i) the potential SNP effect on genic regions or its genotype within the discovery panel, and (ii) the SNP distribution throughout the walnut genome. We selected 66 280 SNPs falling in 23 984 predicted genic regions and featuring mainly mis‐sense or non‐sense genic changes (HIGH_MOD_EFF). These SNPs, which cover 73.8% of all predicted genes in the walnut genome, are of particular interest for marker‐trait association studies, as any change in the amino acid sequence of a protein can potentially have phenotypic effects (Zhang *et al*., 2014). Additionally, we retained 10 933 variants with only homozygous genotypes for the alternative allele and less than three missing data (ALL_HOM), since they might represent fixed alleles in the discovery panel.

Afterward, we combined focal point and tagSNP approaches (see ‘Experimental Procedure’) to select SNPs evenly distributed in the genome, similar to the strategy used in apple (*Malus domestica*) by Bianco *et al*. (2016). This procedure yielded 532 472 SNPs, selected in pre‐defined windows (10 Kb) spread across the whole walnut genome. In this way, we completed the final list of 609 658 entries tiled on the array, assuring full and uniform coverage of the walnut genome and minimal redundancy in genotypic information (Table S2).

The 609K SNPs were spread among 8079 scaffolds covering over 93% (622 Mb) of the *J. regia* reference genome (v1.0). This high‐density SNP array represents a great advance in walnut genetics, based to date on either a handful of markers (e.g. 14 SSRs; Woeste *et al*., 2002) or the genomic variation discovered only in one individual (You *et al*., 2012). Scoring thousands of SNPs evenly distributed along the walnut genome will allow detection of recombination events that occurred during past breeding cycles, providing enough coverage and resolution for association studies and genome prediction.

Based on the minor allele frequency (MAF) distribution, 160 473 SNPs showed a MAF value within the 5%–9% range, 156 359 in 10%–19%, 106 164 in 20%–29%, 101 743 in 30%–39% and 84 919 in the 40%–50% range. A common issue with array‐based technologies is the introduction of ascertainment bias in favour of common alleles during the SNP selection process (Ingvarsson and Street, 2011). The degree of ascertainment bias depends strongly on the size and genetic diversity of the discovery panel. Our re‐sequenced panel was smaller than those used for other SNP arrays in horticultural crops, such as apple (*M. domestica*; Bianco *et al*., 2016), peach (*Prunus persica*; Verde *et al*., 2012) and grapevine (*Vitis vinifera*; LePaslier *et al*., 2013 2013). Based on previous genetic survey with SSR markers, Persian walnut is highly heterozygous (H_o_ = 0.5–0.6; Ruiz‐Garcia *et al*., 2011; Woeste *et al*., 2002). Therefore, we were confident that our selected 27 accessions were covering enough genetic diversity for intra‐species analysis. The heterozygosity estimated using *in silico* SNP genotypes of our discovery panel was on average 0.26 (Table S1), probably because of the differences in levels of polymorphisms between SNPs and SSRs (Emanuelli *et al*., 2013). Similar values of heterozygosity were observed in other highly heterozygous tree species (De Lorenzis *et al*., 2015; Micheletti *et al*., 2015).

### Validation of the Axiom™ *J. regia* 700K array

We used the new Axiom™ *J. regia* 700K array to genotype 1284 walnut accessions, mostly from the UC Davis WIP (Table S3). In particular, the WIP walnut collection was comprised of (i) 68 parents, including 25 of the re‐sequenced founders and 26 walnut cultivars (e.g. ‘Chandler’, ‘Tulare’ and ‘Howard’), and (ii) 48 full‐sib families from crosses made mostly between 2003 and 2013. The number of seedlings per family averaged 23, with the largest family, ‘CRxIDA’, containing 336 individuals. These progeny originated from a wide cross between the cultivars ‘Chandler’ and ‘Idaho,’ which display very distinct phenotypes for several traits, such as kernel quality and fruit size. In addition, the genotyping panel included ten Italian accessions, notably the cultivar ‘Bleggiana’ and one accession of the landrace ‘Feltrina’ grown in Northern Italy, and six accessions of the landrace ‘Sorrento’ from the Southern Italian region Campania, the primary location of walnut production in the country (Foroni *et al*., 2005).

Twelve samples were removed due to a low call rate (*Quality Control*—QC call rate < 97%). These samples likely failed due to low‐quality DNA or the genetic distance from the discovery panel, as might be the case for ‘VX211’ and ‘RX1’, two hybrids of *J. regia* with *J. hindsii* and *J. microcarpa* respectively, which were released as rootstocks from the UC Davis WIP (McGranahan *et al*., 2010a,2010b).

SNPs were sorted into the six default classes of Affymetrix Power Tools (APT) based on clustering performance and quality‐control measures: Poly High Resolution (PHR), No Minor Homozygote (NMH), Off‐Target Variant (OTV), Mono High Resolution (MHR), Call Rate Below Threshold (CRBT) and Other. (Table 1). Afterward, the PHR SNP category underwent a further filtering step recommended for polyploid species, identifying seven additional categories (see ‘*Experimental Procedure*’; to simplify, we merged AAvarX and AAvarY into AAvar, BBvarX, and Y into BBvar, and ABvarX and Y into ABvar; Table 1). This additional step was performed because walnut has undergone a recent whole‐genome duplication (Luo *et al*., 2015), and the presence of paralogous loci may alter the position of the genotyping clusters and, therefore, introduce genotyping errors (Bassil *et al*., 2015).

**Table 1:** Summary of the number of variants per SNP quality category and SNP selection mode, after genotyping a walnut collection with the Axiom™ *J. regia* 700K array

SNP selection type	AAvar	ABvar	BBvar	HHR	CRBT	MHR	NMH	Other	OTV	PHR
ALL_HOM	69	158	182	1	250	334	2216	2820	1772	3040
HIGH_MOD_EFF	385	423	388	4	1432	1647	9401	3986	923	47 691
INFPs	5066	7159	5413	102	24 764	25 718	73 117	91 597	10 802	288 798
Total	**5520**	**7740**	**5983**	**107**	**26 446**	**27 699**	**84 734**	**98 403**	**13 497**	**339 529**
%	0.9	1.3	1.0	0.0	4.3	4.5	13.9	16.1	2.2	55.7

AAvar, AAvarianceX and Y; ABvar, ABvarianceX and Y; ALL_HOM, all homozygous in the discovery panel; BBvar, BBvarianceX and Y; CRBT, Call Rate Below Threshold; HHR, HomHomResolution; HIGH_MOD_EFF, mis‐sense and non‐sense SNPs; INFPs, SNPs in focal points; MHR, Mono High Resolution; NMH, No Minor Homozygote; OTV, Off‐target Variant; PHR, Poly High Resolution.

The top three SNP categories were PHR (55.7%), Other (16.1%) and NMH (13.9%). PHR SNPs are polymorphic with good cluster resolution and high‐quality control measures, and are usually recommended for downstream analysis. The class ‘Other’ included low‐quality SNPs, which showed one or more cluster properties below thresholds and, therefore, did not convert well on the array. As with the PHR SNPs, the NMH showed good clustering performance but did not present any minor homozygous genotypes. A small proportion of variants (4.5%) was monomorphic (MHR) in the genotyping panel, which likely indicates false‐positives due to sequence errors in the discovery panel. The percentage of walnut variants falling into the more thoroughly defined class of PHR SNPs was higher than the one reported for the *CicerSNP* array (26%; Roorkiwal *et al*., 2018), but smaller than the conversion rate of the Apple480K SNP array (74%; Bianco *et al*., 2016) or the Maize 600K array (92%; Unterseer *et al*., 2014). However, the Apple480K SNP array was based on a 6 μm features array technology, which is likely more appropriate for species with duplicated genomes as apple and walnut. On the other hand, in maize the variants incorporated into the final 600K SNP chip were selected after the development and validation of two screening arrays, where only 46.3% of the 1.2 m total variants were classified as PHR.

SNPs identified in predicted genic regions had the highest rate of conversion into PHR (72%) and NMH (14%) polymorphisms. This is consistent with previous observations in peach and apple in which genic SNPs were also the best performers due to the lower level of undetected polymorphisms in the sequences flanking the target SNP (Verde *et al*., 2012). The conversion rate into PHR SNPs dropped to 54% for those SNPs selected in focal points (INFPs) and to 28% for the ALL_HOM SNPs (Table 2). A significant proportion of ALL_HOM and INFPs polymorphisms were classified as ‘Other,’ ‘HomHom Resolution (HHR)’, or ‘CRBT’, confirming the lower conversion rate of non‐genic variants relative to SNPs in coding regions (Chagné *et al*., 2012). Interestingly, 16% of the ALL_HOM SNPs fell into the OTV class, indicating the possible presence of undetected genetic variability in the flanking regions.

**Table 2:** Summary of the main genetic statistics and missing rate in the SNP sets of PHR, robust PHR and robust NMH + OTV

SNP class	N. Markers	Missing rate	MAF	Het
PHR	339 529	0.003	0.21	0.3
Robust* PHR	141 231	0.002	0.24	0.34
Robust* NMH + OTV	49 974	0.002	0.04	0.08

Het, observed heterozygosity; MAF, Minor Allele Frequency; NMH, No Minor Homozygote; OTV, Off‐target Variant; PHR, Poly High Resolution.

* No Mendelian errors across all trios and duos of the UC Davis WIP.

When considering only PHR SNPs, the genotype reproducibility was higher for the biological replicates (99.2%) than for the technical replicates (97.6%) of ‘Chandler’. The lower concordance among technical replicates can be explained by low quality DNA, as is also suggested by their higher missing rate (0.39% on average) compared to the biological replicates (0.16%). Moreover, we also compared the genotype data of the Axiom™ *J. regia* 700K array with the *in silico* variant calls from the high‐depth re‐sequencing of the 27 founders. The genotype concordance was 93% on average, with a minimum of 90.7% for accession ‘UC‐85‐043‐1’ and a maximum of 94.21% for the cultivar ‘Hartley’. The SNPs showing the highest number of genotypic errors were those classified as ‘Other’, confirming the low‐quality and poor informativeness of these variants. Considering only the 339K PHR SNPs, the concordance between genotypes from *in silico* calls and the Axiom™ *J. regia* 700K array increased to 99% on average, which proves the reliability of these markers for downstream genetic analysis.

### WIP pedigree reconstruction

Since its establishment in late 1948, the UC Davis WIP has worked to address the needs of California walnut growers by emphasizing yield, harvest date, kernel colour and in‐shell traits. All controlled crosses made in the WIP have been meticulously registered in a historical pedigree, which was used recently to estimate breeding values (EBVs) for four important traits in the WIP, such as lateral fruit bearing and kernel colour (Martínez‐García *et al*., 2017). However, the WIP historical pedigree still contains unknown relationships and may include errors that could have occurred during different phases of the breeding program.

**Figure 2:**
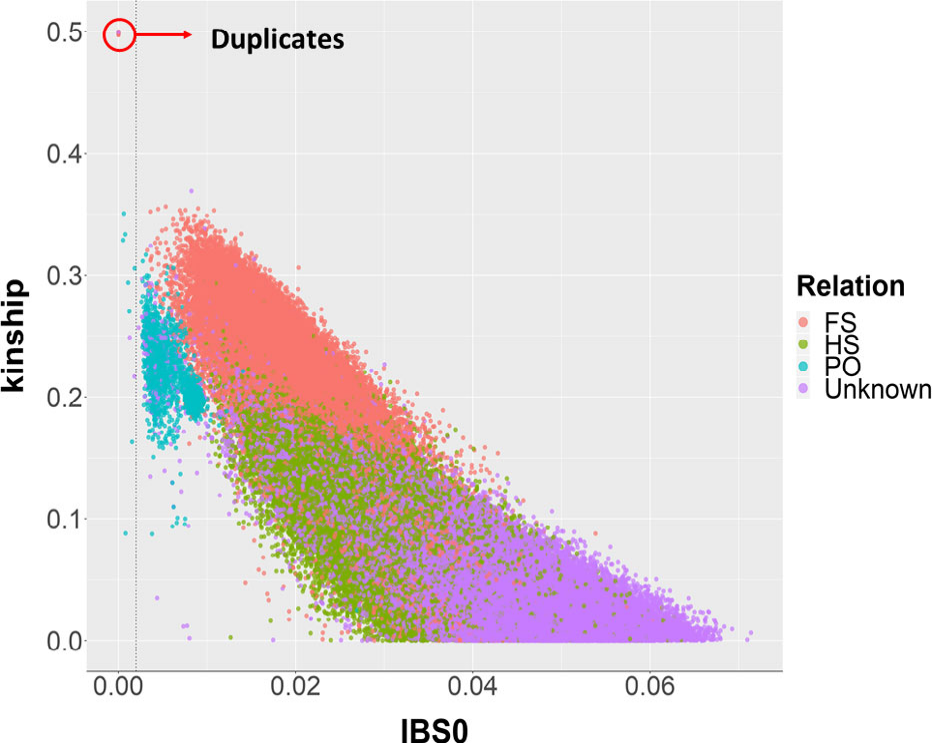
Values of the kinship coefficient and IBS0 for each pairwise comparison among individuals of the UC Davis WIP. Dots are coloured according to four degree of relatedness in the historical WIP pedigree: PO, parent‐offspring; FS, full‐sibs; HS, half‐sibs; Unknown, not recorded relationship (see colour legend). Pairs of genetically identical individuals are marked as duplicates.

In this regard, we estimated the coefficient of kinship (k) for all pairwise comparisons between individuals (Manichaikul *et al*., 2010) by using a final set of 44 738 PHR SNPs, filtered for missing rate (>20%), MAF (< 5%) and linkage disequilibrium (LD > 0.25). In Figure 2, the inferred kinship coefficients are plotted against the estimated proportion of zero identical‐by‐state (IBS0) in four kinds of historical recorded relationships: (i) known parent‐offspring (PO); (ii) known full‐sibs (FS); (iii) known half‐sibs (HS); (iv) unknown relationship. Twenty‐one pairs showed a kinship coefficient higher than 0.45, which indicates genetically identical individuals. ‘Ashley’ and ‘Payne’ were among these pairs, clarifying the controversial high similarity in phenotype and phenology between these two varieties, suspected to be clones (Dangl *et al*., 2005). The remaining duplicates can arise from mislabelling errors during either sample collection or propagation in the orchards.

As shown in Figure S1, *k* ranged between 0.16 and 0.35 for 95% of the known FS and PO relationships. Therefore, we considered all pairwise relationships with *k *≥* *0.16 to be first‐degree relatives. Within the newly defined subset of first‐degree relatives, we observed a maximum value of IBS0 equal to 0.03 among known PO relatives, which was used as a threshold value to distinguish parent‐offspring (IBS0 ≤ 0.03) from full‐sib pairs (IBS0 > 0.03). In this way, we identified both new parents for 17 individuals. In particular, ‘Eureka’ and ‘Payne’ were confirmed as parents of the cultivar ‘Marchetti’, providing genetic evidence for the phenotypic similarity between these walnut varieties, as already predicted by Harold I. Forde, one of the pioneer plant breeders of the UC Davis WIP (Tulecke and McGranahan, 1994). A first‐degree relationship of ‘Waterloo’ with both ‘Eureka’ and ‘Scharsch Franquette’ was also identified. As reported by Tulecke and McGranahan (1994), ‘Waterloo’ was discovered around 1934 among ‘Eureka’ seedlings in California, and is a ‘Eureka’‐type for several traits. The pedigree reconstruction with molecular markers also allowed the identification of 25 mis‐recorded female parents in six families, now corrected for 24 individuals. In addition, ‘Scharsch Franquette’ showed a new and unrecorded first‐degree relationship with the cultivar ‘Cascade’. The characterization of ‘Scharsch Franquette’ with microsatellite markers previously demonstrated its full identity with the original French cultivar ‘Franquette’, which was the primary commercial walnut cultivar in California at the beginning of last century (Dangl *et al*., 2005).

The historical WIP pedigree included 18 open pollinated individuals. We were able to identify a male parent for eight of these. In addition, male parents were determined for three crosses with a previously unknown male parent. We also discovered new male parents for 15 individuals, most likely due to inadvertent outcrossed pollen. Finally, we were able to confirm at least one parent for 1189 individuals and to identify 151 new PO relationships.

**Figure 3:**
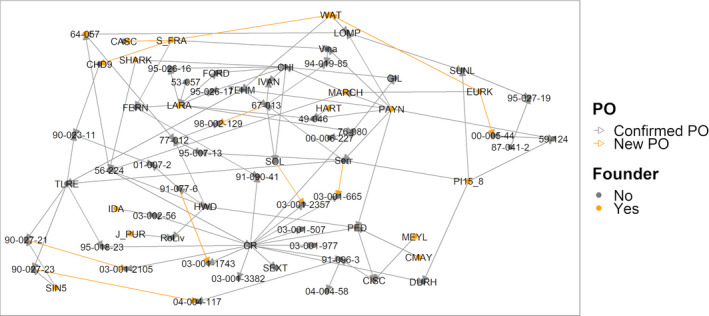
Parent‐offspring relationships of Persian walnut cultivars and the WIP parents genotyped with the Axiom™ *J. regia* 700K array. Orange vertices represent new PO relationships, while orange edges indicate the individuals included in the SNP discovery panel.

We ran the Mendelian inheritance test for all new PO relationships, and defined trios or duos by assigning, respectively, two parents or one parent to a sample when less than 5% of the SNPs were inconsistent with Mendelian inheritance. All new PO relatives were confirmed. The final reconstructed pedigree includes 1225 unique individuals and represents a robust panel to discover marker‐trait associations and predict individual genomic performance for traits of relevance in walnut (Figure 3).

### Genetic diversity

Poly High Resolution, NMH and OTV variants are of particular interest for downstream genetic analysis, such as linkage map construction, GWAS and the study of genetic diversity in walnut germplasm collections. While PHR SNPs are generally recommended because of their well‐distinguished clusters, NMH and OTV may indicate either genotyping errors or genomic regions with unusual genetic patterns, such as deletions or tri‐allelic polymorphisms (Crooks *et al*., 2013). For instance, purifying selection against alleles with unfavourable phenotypes for traits of interest in the UC Davis WIP may explain the lack of the minor homozygous of the NMH SNPs. On the other hand, the OTVs are SNP sites with reproducible and uncharacterized variation in the hybridization probes that can provide additional information on the genetic diversity of the genotyping panel. The same applies to the AAvar, ABvar and BBvar SNPs, which generally show variations either in signal intensity or the contrast of the probes.

By running the Mendelian inheritance test, we defined a new list of 141 231 PHR SNPs with zero Mendelian inconsistencies across all trios and duos tested. In addition to this new set of ‘robust PHR’ SNPs, a new set of ‘robust NMH + OTV’ SNPs was created by including all NMH (45 344), OTV (152), AAvar (1380), ABvar (2192) and BBvar (906) polymorphisms showing no Mendelian errors. In this way, we removed most of the SNPs with an excess of genotyping errors.

We evaluated the missing rate and basic metrics of genetic diversity for PHR, robust PHR and robust NMH + OTV polymorphisms separately. As shown in Table 2, the missing rate was very low (< 3%) in all three SNP sets. Low missing rate is an advantage of SNP array technologies over genotyping‐by‐sequencing (GBS), which generally generates a high‐level of missing data (Rasheed *et al*., 2017). In contrast, the values of MAF and heterozygosity were different among the three SNP subsets (Table 2). The robust PHR variants showed the highest value of MAF, most likely because of the removal of genotyping errors with the Mendelian inheritance test (Figure S2). Instead, NMH + OTV polymorphisms were enriched in low‐frequency alleles (MAF < 0.1; Figure S2), likely resulting from uncommon genetic diversity.

Differences between the three SNP categories also were observed for the inbreeding coefficient (*F*_*IS*_) values of WIP families with more than five seedlings each. *F*_*IS*_ was negative when estimated with PHR (−0.015) and robust PHR (−0.028) and positive when only NMH + OTV (0.032) were considered. This can be explained by the fact that, while PHR variants capture most of the genetic diversity throughout the genome, the NMH and OTV polymorphisms might target specific genomic regions with excess homozygosity due to past selection within the UC Davis WIP. Moreover, the values of *F*_IS_ and heterozygosity for the two PHR classes may derive from bias towards intermediate allele frequencies, commonly introduced by the filtering steps during array development (Unterseer *et al*., 2014). However, the UC Davis WIP showed an overall low level of inbreeding (Figure S3), as previously reported by Martínez‐García *et al*. (2017).

**Figure 4:**
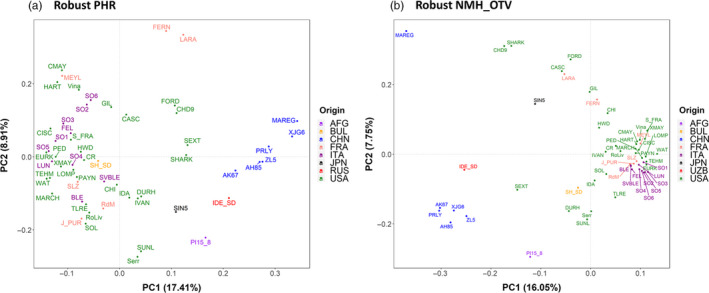
Genetic relationships among 56 walnut cultivars/accessions of different origin (AFG, Afghanistan; BUL, Bulgaria; CHN, China; FRA, France; ITA, Italy; JPN, Japan; UZB, Uzbekistan; USA, United States). PC1 and PC2 were calculated using robust PHR (a) and robust NMH + OTV (b). The variance proportion explained by each PC is shown in parentheses along each axis.

**Figure 5:**
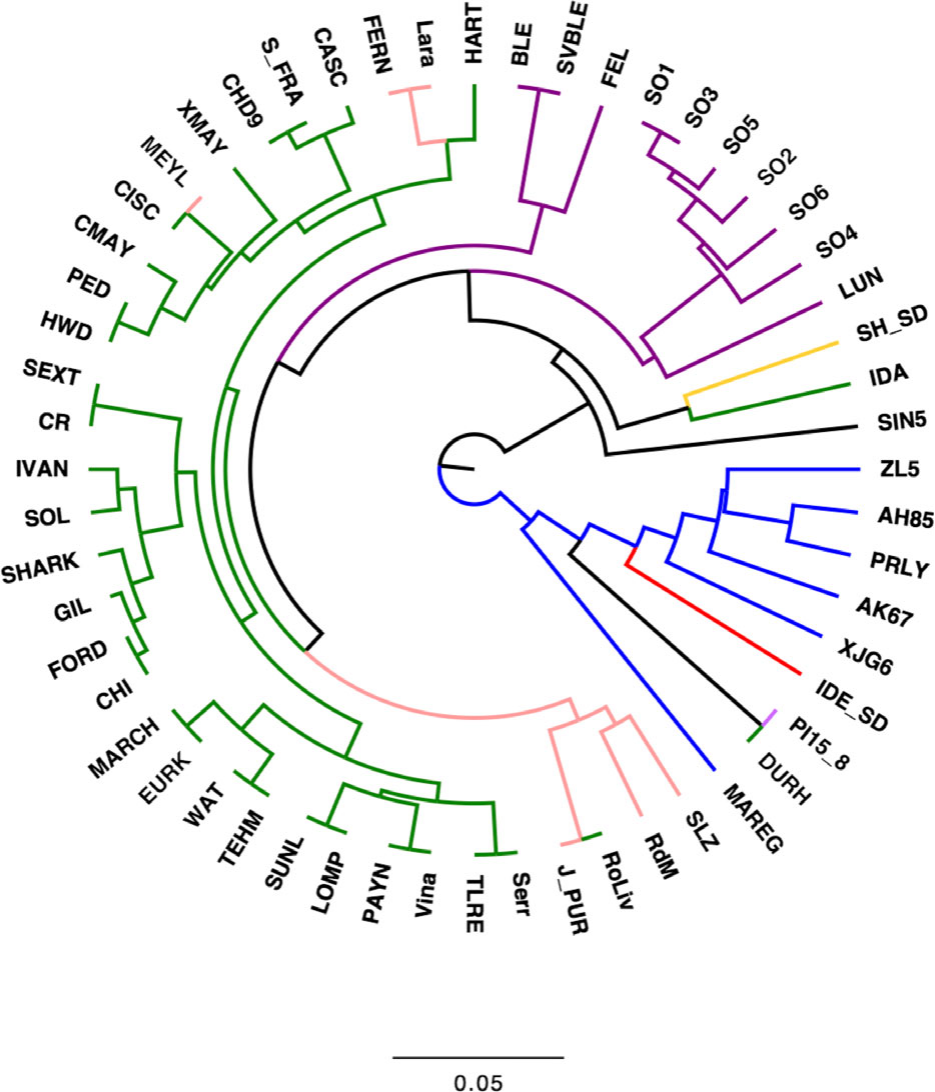
Unrooted UPGMA dendrogram of 56 walnut cultivars/accessions generated using unweighted pair group method with arithmetic mean cluster analysis (UPGMA) based on genetic distance. Branches are coloured according to the geographical origin: green = USA (most from California); pink = France; blue = China; red = Uzbekistan; black = Japan; purple = Italy, yellow = Bulgaria; violet = Afghanistan (only PI15_8).

Besides describing the genetic diversity within the UC Davis WIP, we performed Principal Component Analysis (PCA) to explore the genetic variability among walnut cultivars of different origin, mostly from the United States (California), China, France and Italy (Table S3). Considering only the robust PHR variants, the first two principal components (PCs) account for 26.55% of the total variability. PC1 separated the Chinese cultivars and varieties from Afghanistan, Japan and Uzbekistan, from the walnut accessions of USA, France, Italy and Bulgaria, which were shown to be genetically more similar. The French cultivars did not group tightly together. ‘Lara’ and its progeny ‘Fernor’ were well separated along the PC2 (Figure 4a). The cultivars ‘Forde’, ‘Chase D9’, ‘Sexton’ and ‘Sharkey’ were located in‐between the European‐American and Asian accessions, which is not a surprising result for ‘Sexton’ and ‘Sharkey’: the former is a cross between ‘Chandler’ and the Chinese introduction ‘UC 85‐008’ and the latter might be derived from a seed from China (Tulecke and McGranahan, 1994). This substructure, defined by geographical origin, was also observed in both the PCA with only robust NMH + OTV polymorphisms (Figure 4b) and the clustering analysis (Figure 5). In particular, the latter identified three major clusters: (i) Chinese genotypes together with the accession ‘IDE_SD’, a seedling of the Uzbek cultivar ‘Ideal’ from Central Asia, and ‘PI159568’ from Afghanistan along with its progeny ‘Durham’, (ii) most of the accessions from USA and Europe plus accession UC 85‐043‐1, a Bulgarian seedling of the cultivar ‘Sheinovo’ from Bulgaria; (iii) the Japanese accession ‘Sin5’. This general separation between genotypes from USA‐Europe and Asia is in line with previous surveys comparing walnut genotypes of different origin (Nicese *et al*., 1998; Potter *et al*., 2002). Within cluster *ii*, the varieties from USA and France grouped together, reflecting the plot of genetic relatedness within the WIP pedigree (Figure 3). In particular, within the French subset, ‘Soleze’, ‘Ronde de Montignac’, and ‘*J. purpurea*’ (RA1088) along with its progeny ‘Robert Livermore’ formed a separate group from ‘Lara’, ‘Fernor’ and ‘Meylan’, which were instead admixed with Californian genotypes. The closer relatedness of California and French genotypes can be explained by the series of controlled crosses made in the WIP between French cultivars, chosen for quality and late leafing traits, and the precocious and lateral fruitful varieties of California (Bernard *et al*., 2018; Germain, 1990). The Italian accessions (Figure 5) formed two independent subgroups: all walnuts from Sorrento (Campania Region; South Italy) clustered together, with accessions SO3 showing a first‐degree relationship with all the others according to the IBD analysis (*k *>* *0.16; IBS0 < 0.03). ‘Sorrento’ is more accurately considered as a landrace rather than a variety. It is a mixture of genotypes, showing a heterogeneous phenotypic variability for important commercial traits, such as fruit size and yield. It was common practice in Campania (South Italy) to label plants originating from ‘Sorrento’ seeds as Sorrento (Foroni *et al*., 2005). The cultivar ‘Bleggiana’ and the accession ‘Selvatico_Bleggio’, first‐degree relatives (*k *>* *0.16; IBS0 < 0.03), formed a second subgroup with one of the walnut genotypes from Veneto (North Italy). Interestingly, in cluster *ii* the American accession ‘Idaho’ grouped together with the walnut accessions from Bulgaria. This finding may be explained by the cold‐hardy Carpathian nature of ‘Idaho’, originally kept in the UC Davis collection just as a curiosity for its very large ‘Bijou’ type nuts but with poor quality (Tulecke and McGranahan, 1994).

In conclusion, the Axiom™ *J. regia* 700K array will transform many aspects of walnut genetics. In the UC Davis WIP, availablity of an accurate pedigree will drive the selection of appropriate parents for future artificial crosses, making the best use of genetic variability while avoiding inbreeding depression. At the same time, advanced genetic analysis can now be performed for traits of interest in the WIP to more thoroughly understand their genetic basis and to predict individual performance. The Axiom™ *J. regia* 700K array is also being used in France and Iran to genotype local Persian walnut germplasm collections with interesting phenotypic variation related to biotic and abiotic stresses (Bernard *et al*., 2018). All together, these studies demonstrate that the Axiom™ *J. regia* 700K array is a powerful genomic tool for providing further insight into the genetics of Persian walnut.

## Experimental procedures

### SNP discovery and selection for the Axiom™ *J. regia* 700K array

The discovery panel included 27 walnut accessions identified as major founders of the UC Davis WIP by Martínez‐García *et al*. (2017) (Table S1). Whole‐genome resequencing, variant discovery and pre‐selection were described previously by Stevens *et al*. (2018). Briefly, DNA was extracted after nuclei isolation from adult leaves of each accession. Paired‐end and mate pair libraries were prepared, pooled and sequenced in paired‐end Rapid Run mode on a HiSeq 2500 System (Illumina; San Diego, CA). Reads were aligned onto the *J. regia* reference genome (v1.0) using BWA (v0.7.13) with default parameters (Li and Durbin, 2009; Martínez‐García *et al*., 2016). A multi‐sample VCF file was then produced using SAMtools, and SNPs were identified using the multi‐sample allele calling algorithm implemented in BCFtools (v1.2; Li *et al*., 2009). A first Quality Filter was then applied to remove poorly scoring variants and false‐positive calls with BCFtools (Figure 1). In detail, SNPs were filtered out if (i) they had low‐quality (QUAL ≤ 60, MQ ≤ 20), (ii) read coverage was < 200 or > 4580, (iii) they showed strand bias (all DP4[i] > 1 for i in range 1,4), (iv) less than 75% of the reads used to call the variant had high quality (sum(DP4[i])/DP < 0.75 for i in range 1,4), and (v) they were very rare (AC/AN < 0.05) or very frequent (AC/AN > 0.95).

Variants were further filtered following the recommendations of Affymetrix (Santa Clara, CA; www.affymetrix.com ; Figure 1). In particular, all the variants were submitted to Affymetrix for an *in‐silico* analysis to predict their reproducibility in the Axiom™ array. The following characteristics were evaluated: (i) existence of other polymorphisms within the 35‐bases adjacent to the target SNP; (ii) high sequence similarity between all 16‐mer sequences flanking the SNPs and the rest of the genome (*16‐mer count*); (iii) the predicted probability that the SNP will convert on the array (*P‐convert score*). Entries with additional predicted variants in both flanking regions, a *16‐mer count *> 300 and a *P‐convert score *≤ 0.6 for both the forward and reverse probes were removed.

The subsequent selection of the SNPs to be tiled on the array involved the following steps:

1. SNP effect on predicted genes was assessed with SNPeff (Cingolani *et al*., 2012), and all SNPs featuring mainly a mis‐sense and non‐sense changes on a gene (*HIGH* and *MODERATE* effects) were selected.
2. SNPs with homozygous genotypes for either the reference or the alternative allele, and with no more than three missing values were selected (*ALL_HOM*).
3. A focal point/tagSNP approach similar to the one used in previous SNP arrays (Bianco *et al*., 2016) was adopted to reach the target number of SNPs to be tiled in the array. In particular, SNPs were selected from pre‐defined windows (10Kb) centered around focal points spread across the whole walnut genome. SNPs showing either all heterozygous or all homozygous genotypes in the discovery panel and those with more than six missing values were discarded. A tagSNP approach was then applied to minimize redundancy by discarding all SNPs too strongly correlated with each other in a focal point window. A LD value of *r² *= 0.81 was used as a threshold.

Contrary to other SNP arrays (Bianco *et al*., 2016), A/T and G/C SNPs were retained, even though they require two probes (one probe per allele) and, therefore, reduce the effective available space on the array.

### Validation of SNPs by genotyping

A total of 1284 walnut accessions of the UC Davis WIP and ten Italian walnut accessions were genotyped using the Axiom™ *J. regia* 700K SNP array (Table S2). Young leaves were collected, freeze‐dried and pulverized. DNA was extracted with the E‐Z 96^®^ Plant DNA Kit (Omega Bio‐tek; Norcross, GA). In particular, the initial lysis incubation was performed with the addition of proteinase K and polyvinylpyrrolidone (PVP) and extended to 90 min. Up to 50 μL of DNA (≥ 20 ng/μL) from each sample were submitted to Affymetrix for genotyping using the Affymetrix GenTitan platform. The same DNA sample from the cultivar ‘Chandler’ was included twelve times as technical replicate (one per plate). Four independent DNA extractions were also performed from leaves of the same ‘Chandler’ tree, and were considered to be biological replicates.

The raw hybridization intensity data were processed with the Affymetrix^®^ Genotyping Console™ software (GTC) (v4.2) for clustering and genotype calling. Samples with a Dish Quality Control (DQC) value < 0.82 and call rate < 0.97 were excluded from further genotyping analysis. APT (v1.19.0) was used for post‐processing the results of GTC and classifying the SNPs into six major classes: PHR, NMH, OTV, MHR, CRBT and Other. A second run of filtering further processed the PHR SNPs, identifying seven additional subclasses to a new, more thoroughly defined PHR class (Affymetrix, 2011): BB variance X and, BBvarY, ABvarX and ABvarY, AAvarX and AAvarY and HHR.

### WIP Pedigree reconstruction

IBD statistics between each pair of individuals were estimated with the *Kinship‐based Inference for Genome‐wide association studies* (KING) framework (Manichaikul *et al*., 2010) implemented in the R package SNPRelate (v 1.10.2; Zheng *et al*., 2012). The KING‐robust algorithm was applied, providing a robust relationship inference in the presence of population structure. A pruned set of PHR SNPs (LD threshold = 0.25, a sliding window of 50 kb) was used, after filtering for missing rate (> 0.2) and MAF (< 0.05). Pairs of accessions were considered to be genetically identical when the kinship coefficient (*k*) was higher than 0.45. Known pedigree relationships were used to calibrate the kinship coefficients, and define novel parent‐offspring pairs. First, all pairwise relationships were considered to be first‐degree relatives if *k *≥* *0.16. The proportion of SNPs with zero IBS0 was then used to further distinguish parent‐offspring (IBS0 ≤ 0.03) from full‐sib pairs (IBS0 > 0.03). The proportion of SNPs consistent with Mendelian inheritance for all identified parent‐offspring relationships was estimated using the function *mendel* implemented in Plink (v1.90b5.3; Chang *et al*., 2015). New trios or duos were defined by assigning, respectively, two parents or one parent to a sample when < 5% of SNPs were inconsistent with Mendelian inheritance.

### Genetic diversity analysis

Missing rate, MAF, heterozygosity and fixation index (*F*_*IS*_) were calculated separately for the PHR, robust (no Mendelian errors) PHR and robust ‘NMH + OTV’ SNPs (including AAvarX, AAvarY, ABvarX, ABvarY, BBvarX and BBvarY), using the commands *freq, freqx* and *missing* in PLINK (v1.90b5.3; Chang *et al*., 2015). Also, PCA and clustering analysis were carried out on a subset of 56 cultivars/accessions from USA (30), Italy (10), China (6), France (6), Afghanistan (1), Bulgaria (1), Japan (1) and Uzbekistan (1) (Table S2). In particular, PCA was performed with the R package SNPRelate, using robust PHR and robust NMH + OTV SNPs, already filtered for missing rate (> 0.2) and MAF (< 0.05). Only robust PHR SNPs were used to produce a dendrogram based on genetic distance values applying the unweighted pair group method with arithmetic mean cluster analysis (UPGMA; Sneath and Sokal, 1973), implemented in the software PHYLIP (v3.695; Felsenstein, 2005). The dendrogram plot was drawn with FigTree (v1.4.3) (http://tree.bio.ed.ac.uk/software/figtree/).

## Conflict of interest

The authors declare no conflict of interest.

## Supporting information


**Figure S1** Distribution of the coefficient of kinship within the four degrees of relationship.
**Figure S2** Frequency polygons for MAF values in the three SNPs sets of PHR, robust PHR, and robust NMH + OTV.
**Figure S3** Fixation index in families of the UC Davis WIP with a minimum of 5 progeny each. The family ‘Other’ includes walnut cultivars, parents and small families (N individuals < 5).
**Table S1** List of the walnut accessions included in the SNP discovery panel.

**Table S2** List of the 609 658 SNPs tiled on the Axiom™ *J. regia* 700K SNP array. Details about the SNP position, alleles and classification after genotyping are provided.

**Table S3** List of the walnut accessions genotyped with the Axiom™ *J. regia* 700K SNP array.
