# Supplementary material for: A new genomic tool for walnut (Juglans regia L.): development and validation of the high‐density Axiom™ J. regia 700K SNP genotyping array
Source: Plant Biotechnol J. 2018 Dec 4;17(6):1027–36. doi: 10.1111/pbi.13034 (PMC6523593; doi:10.1111/pbi.13034)

Supporting Information

A new genomic tool for walnut (*Juglans regia* L.): development and validation of the high-density Axiom™ *J.regia*700K SNP genotyping array

Annarita Marrano[1], Pedro J. Martinez-Garcia[1], Luca Bianco[2], Gina M. Sideli[1], Erika Dipierro[2], Charles A. Leslie[1], Kristian A. Stevens[3], Marc W. Crepeau[3], Michela Troggio[2], Charles H. Langley[3] and David B. Neale[1]

Table S1. List of the walnut accessions included in the SNP discovery panel. For each accession, we reported the geographical origin, the total number of trimmed and mapped reads, the mean coverage depth across the genome after the alignment, if it was genotyped or not with the new Axiom^{TM} *J. regia* 700K array, as well as heterozygosity.

Accession name	Library ID	Total trimmed reads	Total mapped reads	Mean coverage depth (X)	Origin	Genotyped	Heterozygosity*
Sinensis #5	JG0001	464,691,916	462,405,726	68.6	Japan	Yes	0.25
Lara	JG0002	471,646,036	463,426,912	69.3	France	Yes	0.27
Payne	JG0012	486,672,430	483,965,271	73.8	USA	Yes	0.24
Manregian	JG0014	467,308,426	464,239,028	72.5	China	Yes	0.23
J. purpurea	JG0018	477,442,940	475,609,050	72.9	France/Germany	Yes	0.24
Sharkey	JG0026	485,397,442	482,795,180	72.7	USA	Yes	0.34
Meylan	JG0031	474,333,174	472,302,102	73.8	France	Yes	0.21
PI159568	JG0032	561,768,302	554,289,734	81.5	Afghanistan	Yes	0.30
Marchetti	JG0041	504,710,080	501,522,686	75.3	USA	Yes	0.24
UC-87-041-2	JG0050	601,830,906	594,611,565	88.1	USA	No	0.24
Conway-Mayette	JG0051	467,683,824	464,949,738	71.1	USA	Yes	0.24
S.Franquette	JG0058	470,725,528	468,591,119	71.3	USA/France	Yes	0.25
UC-64-057	JG0061	525,745,114	522,167,210	78.9	USA	Yes	0.25
UC-85-008	JG0065	475,185,914	472,653,849	72.3	China	Yes	0.26
Idaho	JG0071	477,121,486	473,613,277	72.6	USA	Yes	0.27
Hartley	JG0072	476,759,118	474,018,449	74.8	USA	Yes	0.16
Waterloo	JG0074	482,425,016	480,354,174	72.4	USA	Yes	0.25
UC-85-043-1	JG0166	486,719,778	483,462,788	74.0	Bulgaria	Yes	0.28
UC-91-041-12	JG0169	480,708,608	477,021,468	70.8	China	Yes	0.30
UC-91-013-5	JG0171	469,471,128	466,779,453	72.0	China	Yes	0.26
UC-91-056-9	JG0172	478,598,010	475,607,857	72.9	China	Yes	0.28
UC-91-031-8	JG0173	472,944,198	470,281,088	71.7	China	Yes	0.28
UC-91-028-2	JG0175	494,191,154	491,029,046	75.3	China	No	0.29
Eureka	JG0176	473,436,490	471,551,254	72.7	USA	Yes	0.22
UC-00-005-149	JG0182	475,176,224	473,443,253	72.5	USA	Yes	0.28
UC-07-045-22	JG0184	465,458,078	460,187,256	69.4	USA	Yes	0.27
Soleze	soleze	487,867,152	481,832,051	73.9	France	Yes	0.23

*We estimated heterozygosity using the 609,658 SNPs selected to be tiled on the array.

Figure S1. Distribution of the coefficient of kinship within the four degrees of relationship (PO = parent-offspring; FS = full-sibs; HS = half-sibs; Unknown = not recorded relatedness).

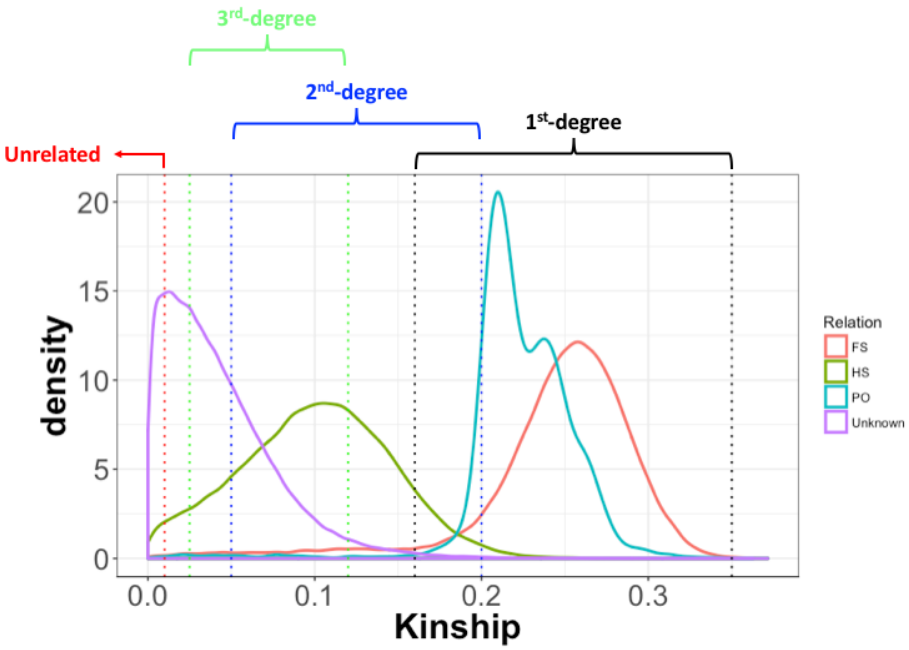

Figure S2. Frequency polygons for minor allele frequency (MAF) values in the three SNPs sets of PHR, robust PHR and robust NMH+OTV.

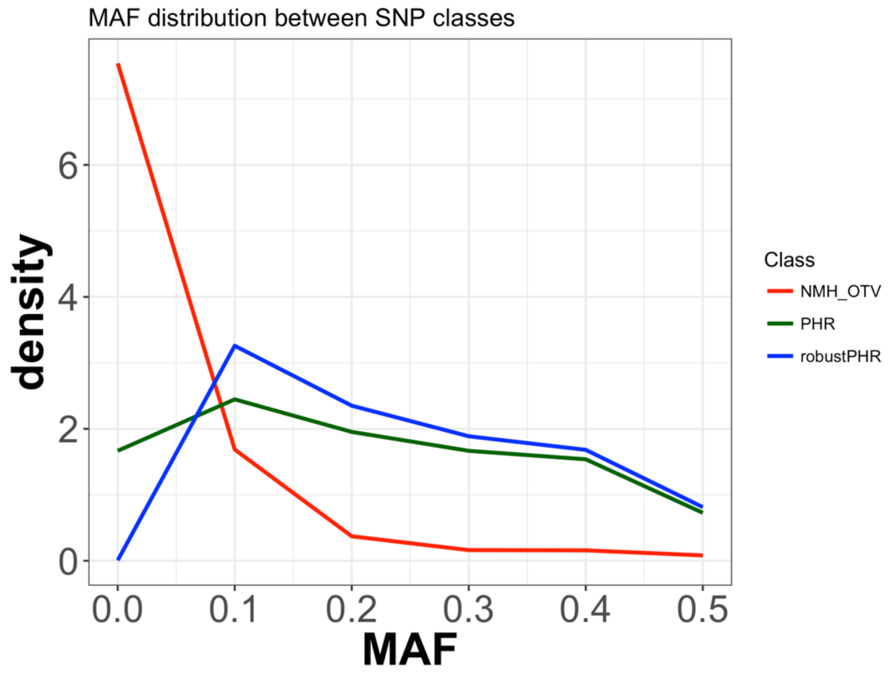

Figure S3. Fixation index in families of the UC Davis WIP with a minimum of 5 progeny each. The family “Other” includes walnut cultivars, parents and small families (N individuals < 5).

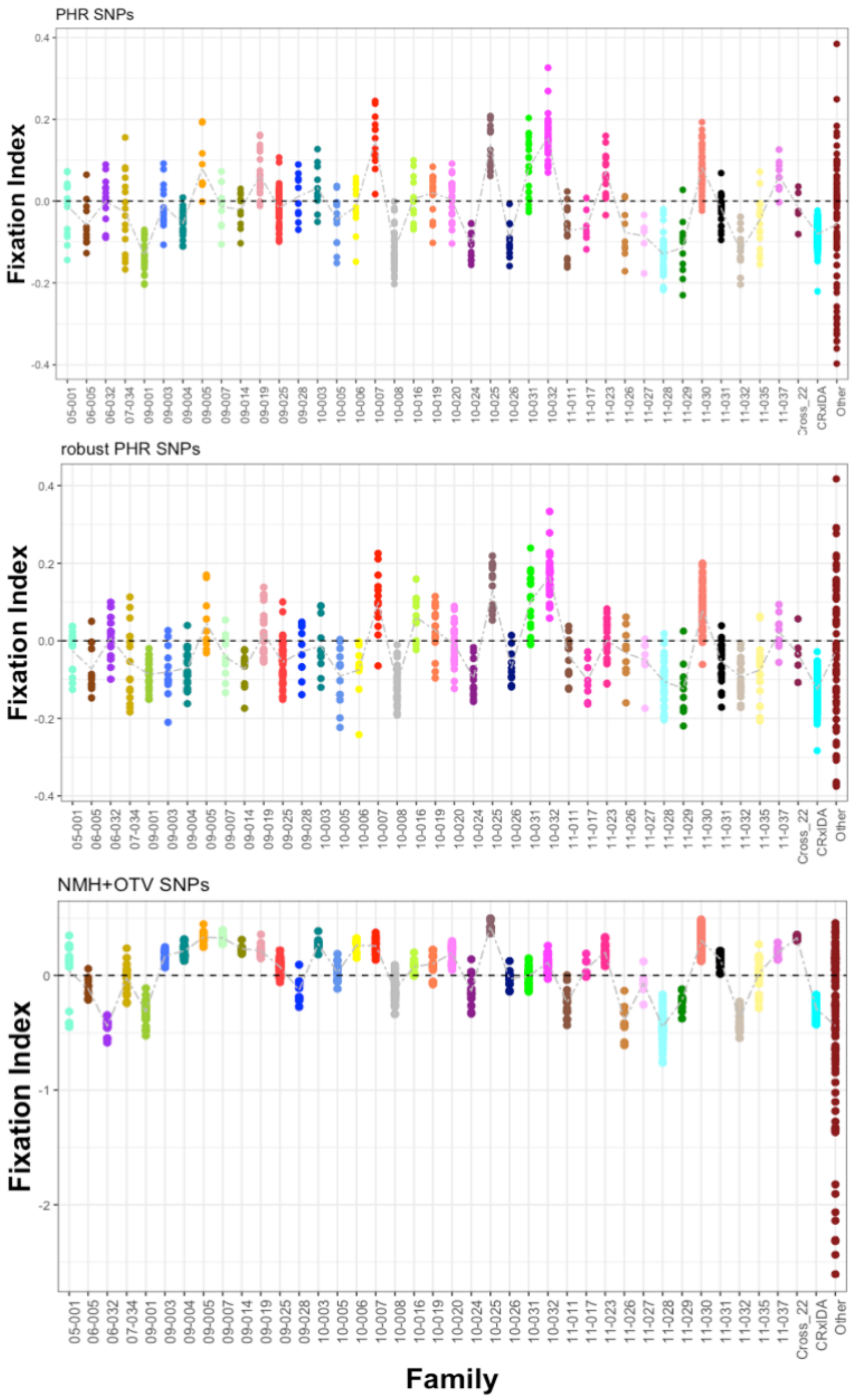

Supplement: Supplementary file 1 — Figure S1 Distribution of the coefficient of kinship within the four degrees of relationship. Figure S2 Frequency polygons for MAF values in the three SNPs sets of PHR, robust PHR, and robust NMH + OTV. Figure S3 Fixation index in families of the UC Davis WIP with a minimum of 5 progeny each. The family ‘Other’ includes walnut cultivars, parents and small families (N individuals < 5). Table S1 List of the walnut accessions included in the SNP discovery panel. [file PBI-17-1027-s002.pdf]
